# Supplementary material for: Engineering human pluripotent stem cells into a functional skeletal muscle tissue
Source: Nat Commun. 2018 Jan 9;9:126. doi: 10.1038/s41467-017-02636-4 (PMC5760720; doi:10.1038/s41467-017-02636-4)
Supplement: Supplementary file 1 — Supplementary Information [file 41467_2017_2636_MOESM1_ESM.pdf]

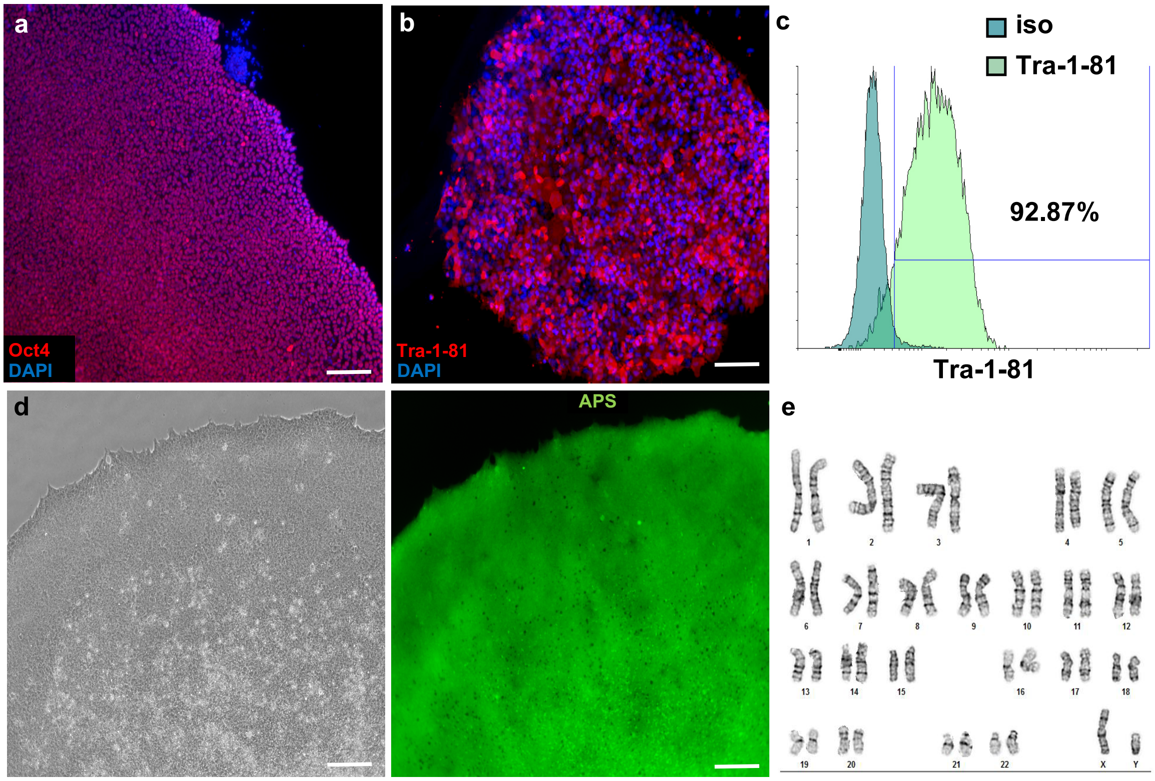

Supplementary Figure 1. Expression of pluripotency markers and karyotype analysis for TRiPSC line. (a) Representative co-staining of Oct4 and DAPI in undifferentiated TRiPSCs. **(b)** Co-staining of Tra-1-81 and DAPI in undifferentiated TRiPSCs. **(c)** Live cell FACS analysis for Tra-1-81^{+} undifferentiated TRiPSCs. Iso, isotype antibody control. **(d)** Alkaline Phosphatase (APS) live staining in undifferentiated TRiPSCs. **(e)** TRiPSCs exhibit normal karyotype. Scale bar, 200μm.

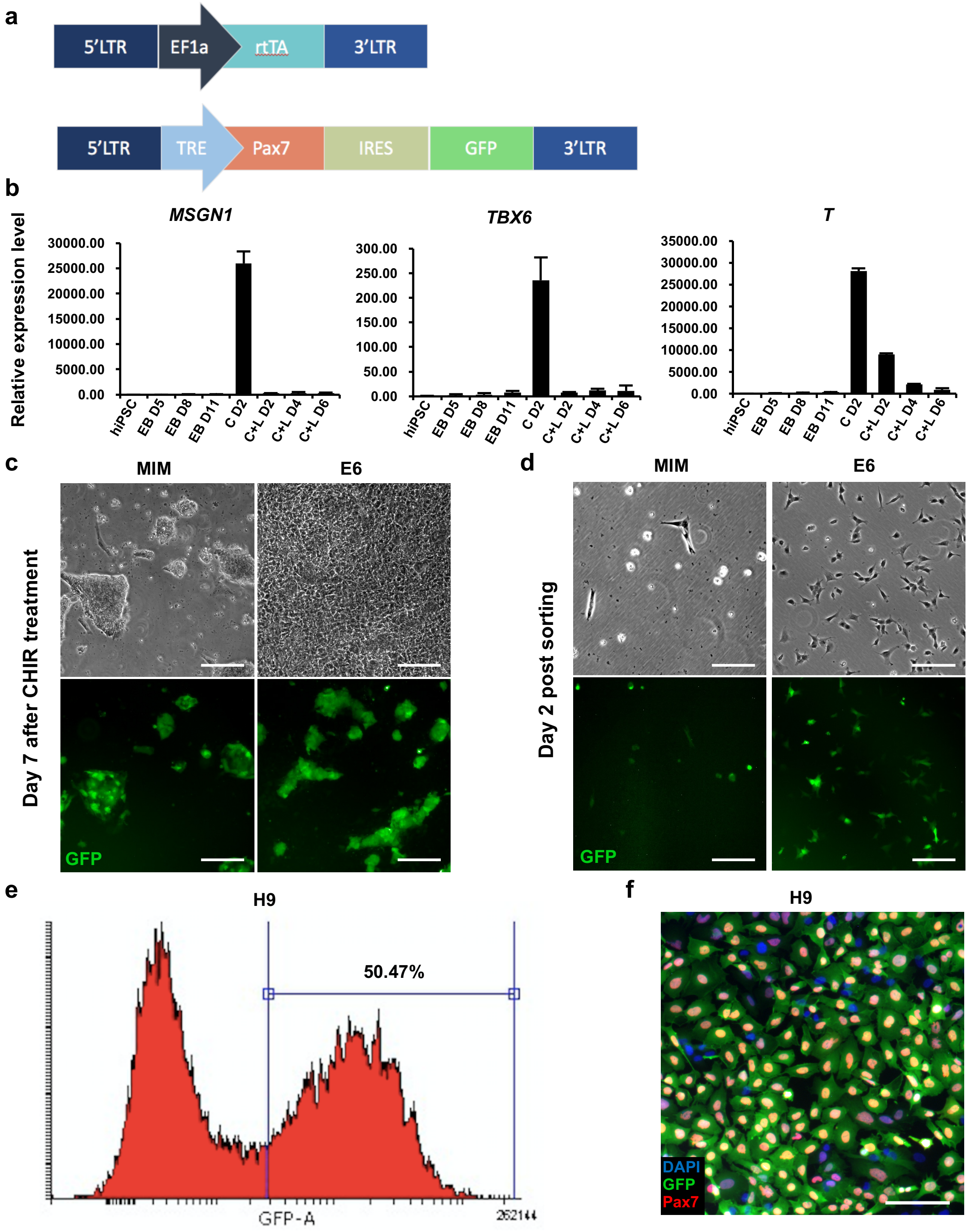

Supplementary Figure 2. Optimizing myogenic progenitor cell derivation. (a) Tet-on lentiviral plasmids used for transient Pax7 induction. **(b)** mRNA expression level of paraxial mesoderm markers, *MSGN1* and *TBX6*, and early mesoderm marker, *T*, under various treatments for mesoderm induction. EB D5/8/11: 5/8/11 days in embryoid body (EB) suspension culture; C D2: 2 days in CHIR99021 (10μM); C+L D2/4/6: 2/4/6 days in CHIR99021 (3μM) + LDN193189 (0.5μM). Expression shown as fold change relative to undifferentiated hPSCs (n=4 samples for hPSC ctrl, n=6 samples from 3 differentiations for other data points). **(c-d)** Comparison of MIM and E6 induction media before (**c**) and after (**d**) sorting for GFP. **(e)** Representative FACS analysis of GFP^+ cells from H9 at sorting. **(f)** Representative immunostaining of GFP and Pax7 in H9 derived progenitors at day 4 post sorting. Scale bar, 50μm. Data are presented as mean ± SEM.

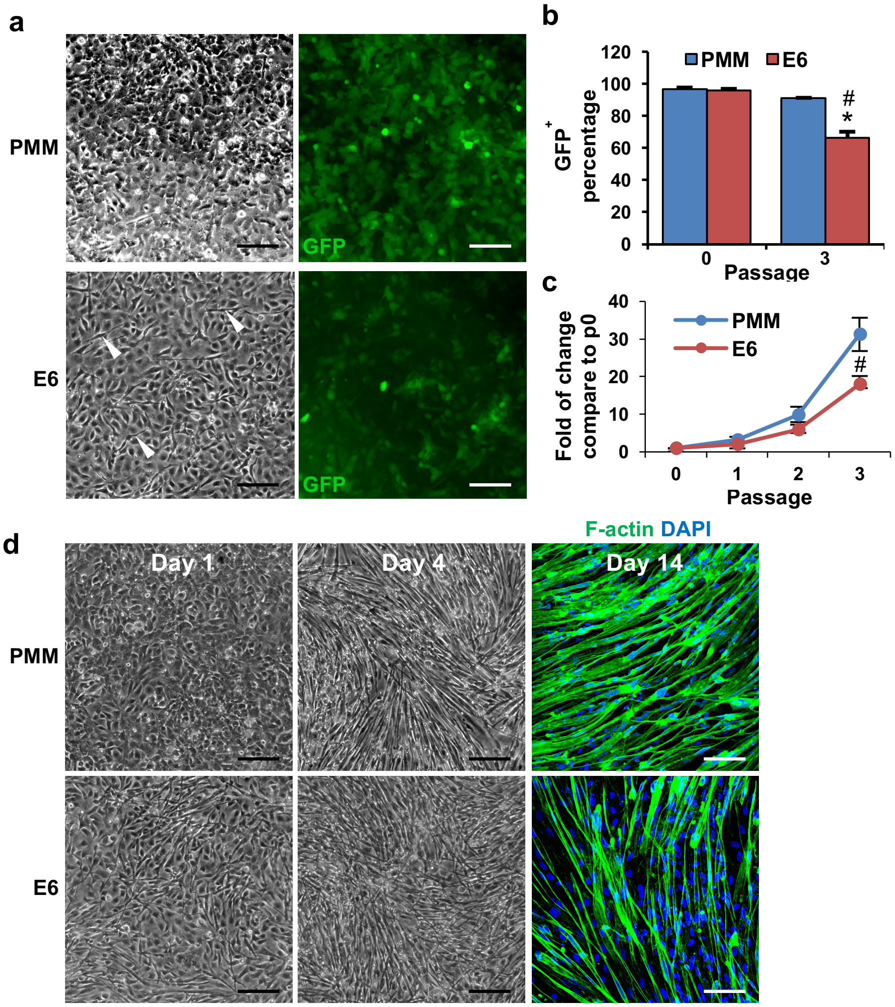

Supplementary Figure 3. Optimizing myogenic progenitor cell expansion. (a) Representative live images of post-sorted GFP$^+$ cells in PMM and E6 expansion media. Arrows indicate cells with spindle-like shape. Scale bar, 100μm. **(b-c)** Quantification of GFP$^+$ cell fractions (**b**) and total cell numbers (**c**) during passaging in different expansion media (*P<0.05 vs. E6 passage 0, #P<0.05 vs. PMM passage 3, Tukey-Kramer HSD test; n = 6 samples from 2 expansions for each condition, data are presented as mean ± SEM). **(d)** Representative images of expanded cells differentiated in monolayers for 1, 4 and 14 days showing higher myotube density in PMM-expanded cells. Scale bar, 100μm.

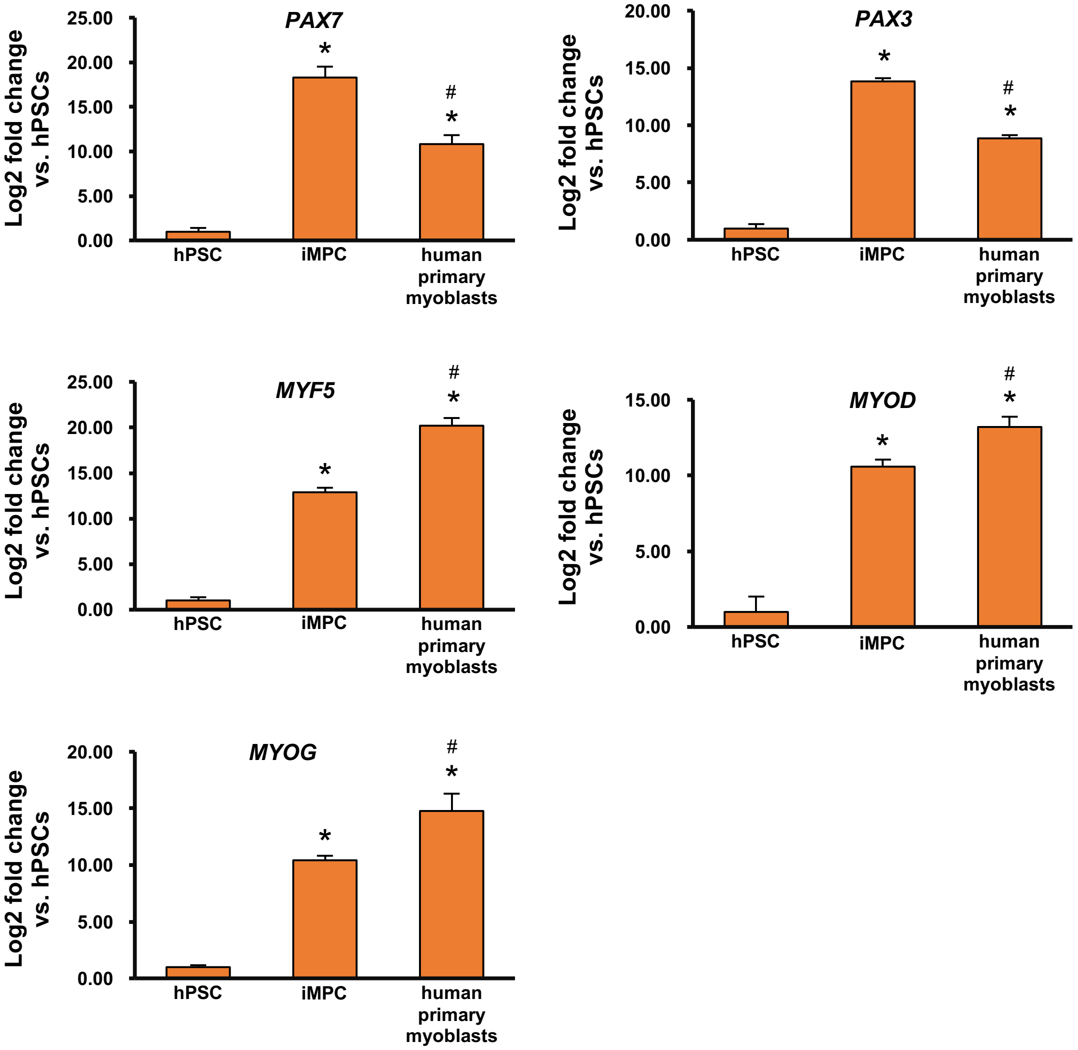

Supplementary Figure 4. Relative mRNA expression levels of *PAX7, PAX3, MYF5, MYOD*, and *MYOG* in undifferentiated hPSCs, iMPCs, and early passaged primary human myoblasts. (*P<0.05 vs. hPSC, #P<0.05 vs. iMPC, Tukey-Kramer HSD test; for hPSCs, n=4 samples from 2 hPSC lines, for iMPCs, n=6 samples from 3 differentiations, for primary human myoblasts, n=4 samples from 2 donors, data are presented as mean ± SEM).

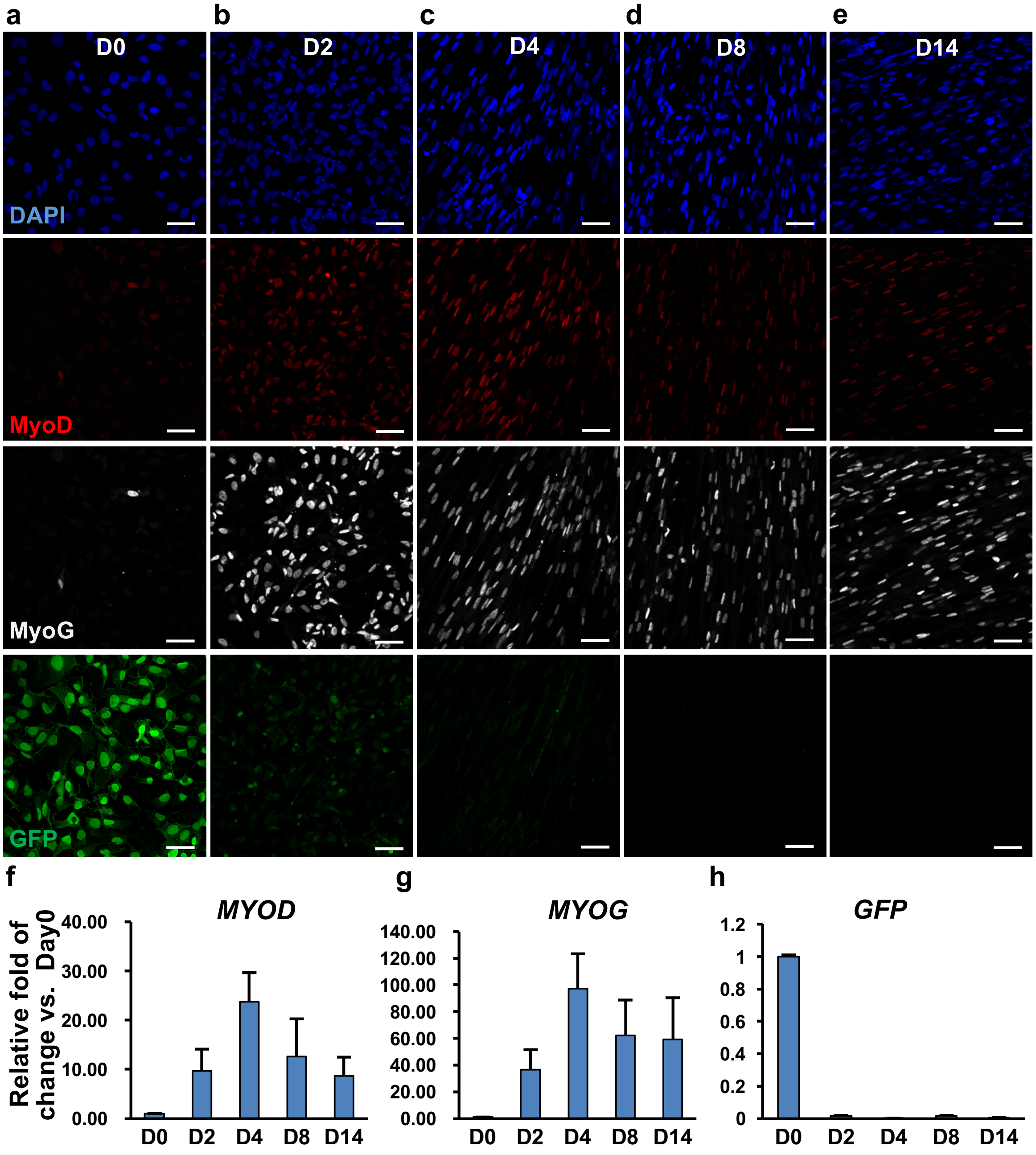

Supplementary Figure 5. Dynamic expression of myogenic markers during 2-week monolayer differentiation of iMPCs. (a-e) Representative immunostaining of MyoD, MyoG, and GFP fluorescence at day (D) 0, 2, 4, 8, and 14 of differentiation. Scale bar, 25μm; **(f-h)** Relative mRNA expression levels of *MYOD, MYOG* and *GFP* during 2-week differentiation (n=4 samples from 2 differentiations, data are presented as mean ± SEM).

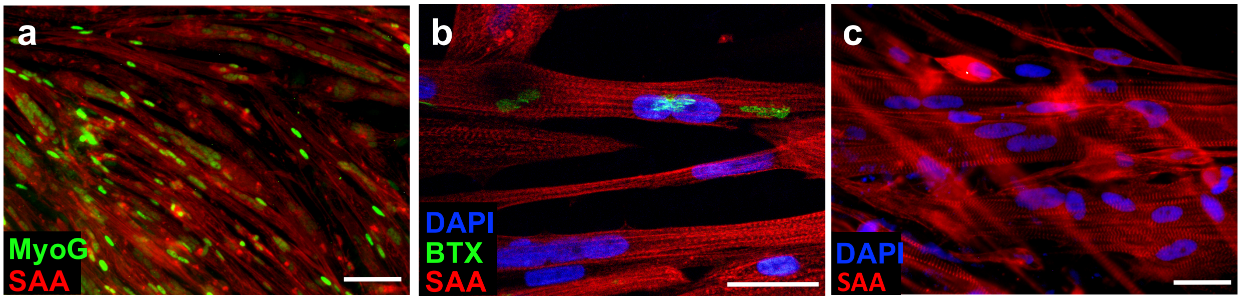

Supplementary Figure 6. Monolayer differentiation of iMPCs. Representative immunostaining of SAA, MyoG, and α-bungarotoxin (BTX) after 2 weeks of monolayer differentiation of iMPCs showing high efficiency of fusion and myogenesis (**a**, scale bar, 100μm), formation of acetylcholine receptors (**b**, scale bar, 25μm), and cross-striations (**c**, scale bar, 25μm).

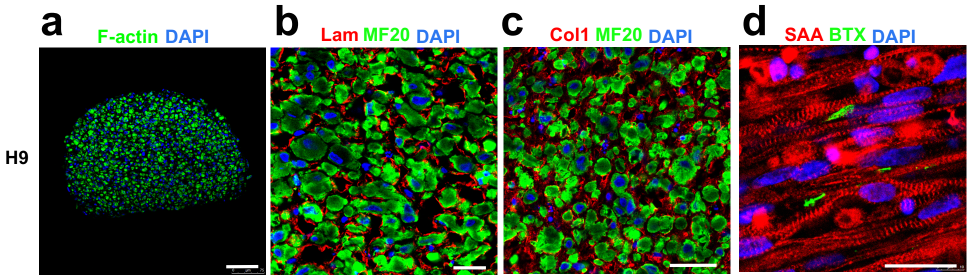

Supplementary Figure 7. Structure of iSKM bundles made from H9 line. (a-c) Representative immunostaining of dense, uniformly distributed, myotubes in bundle cross-section. Scale bars: **a**, 75µm; **b, c,** 25µm. Col 1, collagen I. **(d)** Representative longitudinal section of 2-week bundles showing aligned, cross-striated myotubes with BTX labeled acetylcholine receptors. Scale bars, 25µm.

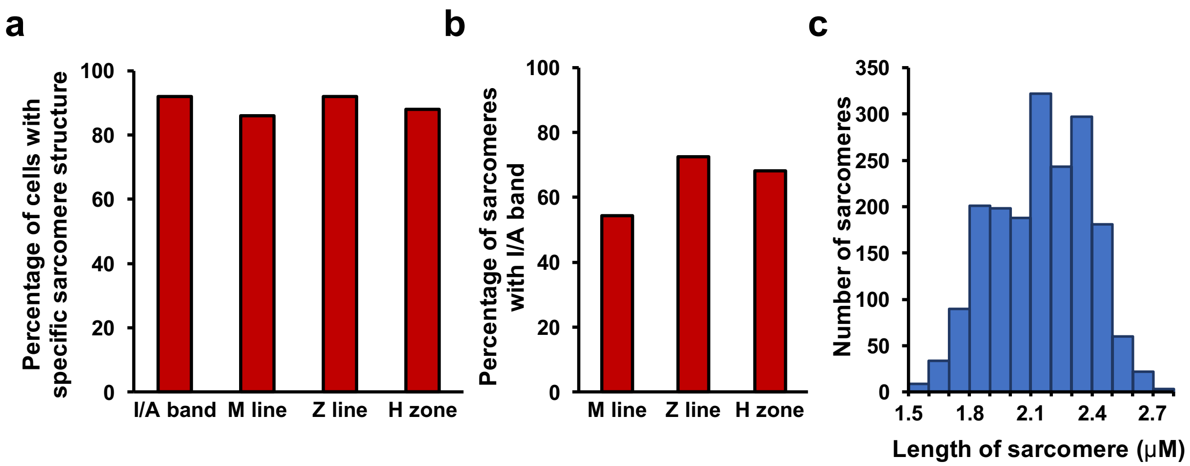

Supplementary Figure 8. Quantification of sarcomeric structures in 4-week differentiated iSKM bundles. (**a**) Percentage of cells with I/A-band, M-line, Z-line and H-zone (n=50 cells). (**b**) Percentage of sarcomeres containing I/A bands that also contain M-line, Z-line, and H-zone (n=1367 sarcomeres). (**c**) Distribution of sarcomere lengths (n=1848 sarcomeres).

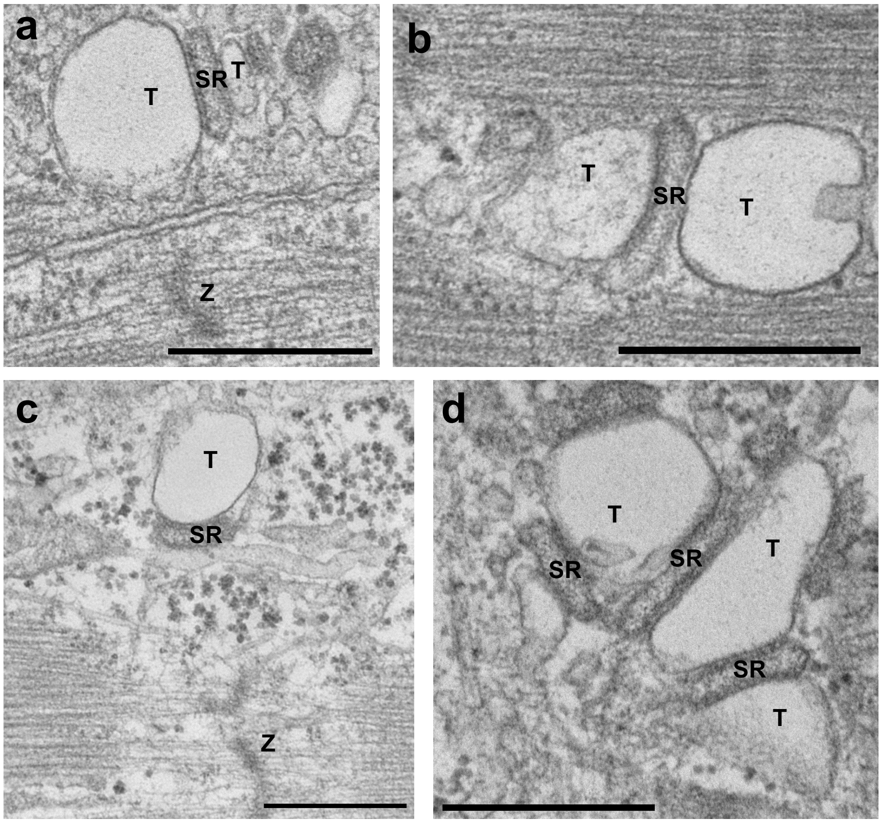

Supplementary Figure 9. Examples of T-SR structures observed in myotubes from 4-week differentiated iSKM bundles. (a,b) Inverted triads. **(c)** Diads. **(d)** multi-junction structure. Z: Z-line; T: T-tubule; SR: sarcoplasmic reticulum. Scale bars, 500nm.

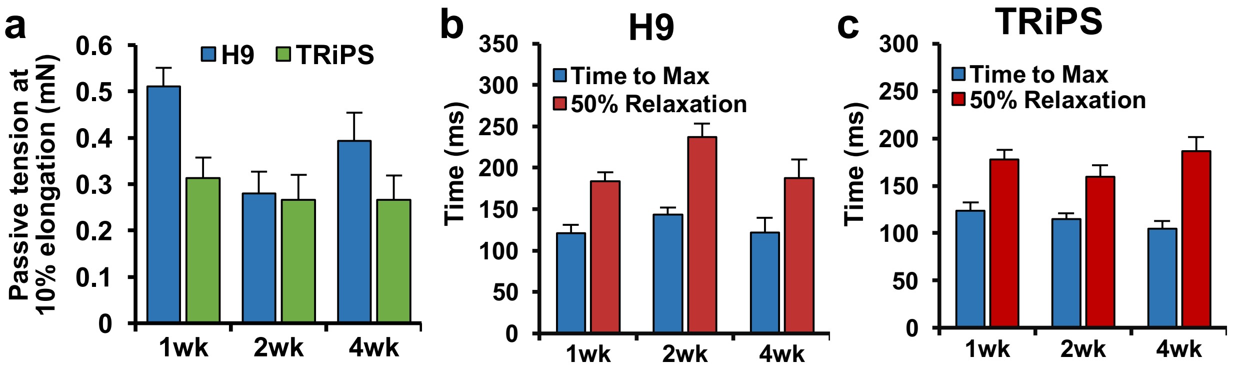

Supplementary Figure 10. Passive mechanical properties and twitch kinetics of iSKM bundles. (**a**) Passive tension of H9 and TRiPS bundles during 4-week culture. (**b**, **c**) Twitch kinetics of H9 (**b**) and TRiPS (**c**) derived iSKM bundles during 4-week culture (n=6-8 bundles per group at each time point, data are presented as mean ± SEM).

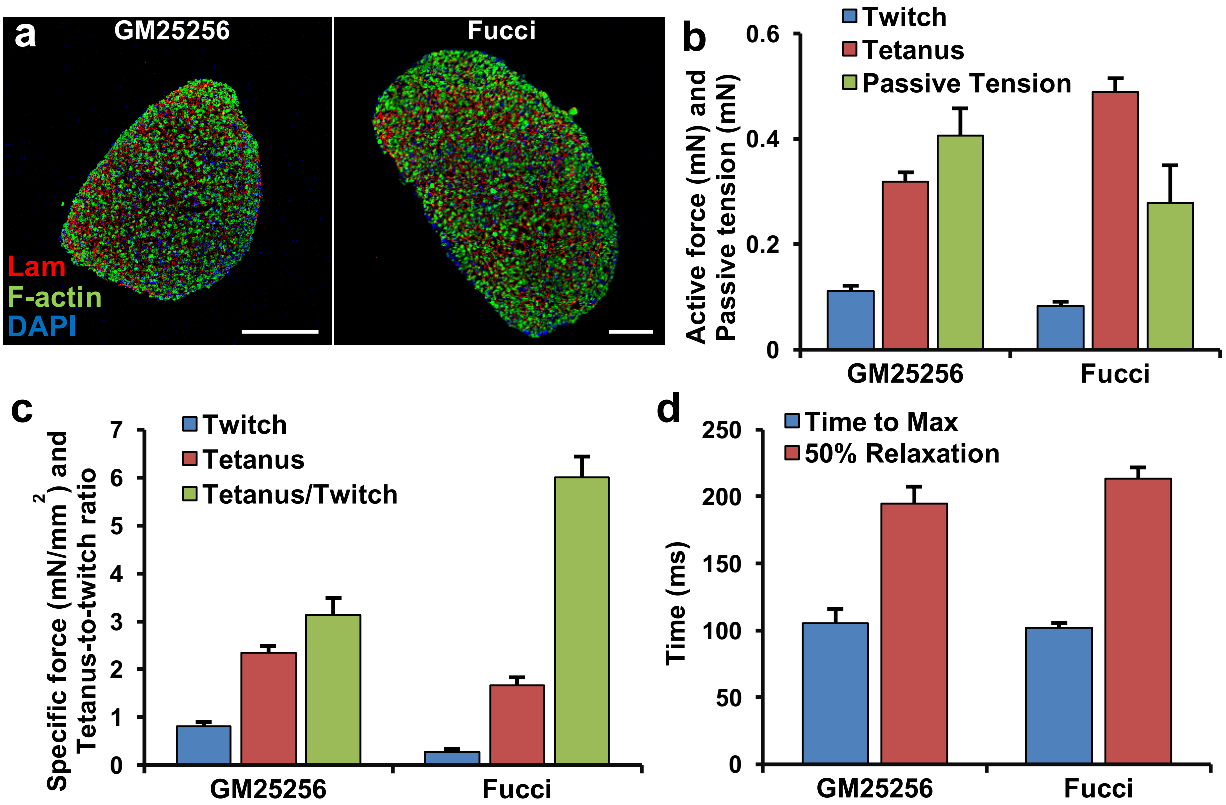

Supplementary Figure 11. iSKM bundles derived from GM25256 and Fucci hPSCs. (**a**) Representative cross-sections of 2-week differentiated iSKM bundles showing dense F-actin$^+$ myofibers surrounded by Laminin (Lam). Scale bar, 100µm. (**b**) Amplitudes of twitch and tetanus (at 40 Hz) and passive tension at 10% elongation generated by GM25256 and Fucci derived iSKM bundles at 2 weeks of differentiation (n=8 bundles for each line). (**c**) Specific forces and tetanus-to-twitch ratios of 2-week differentiated GM25256 and Fucci derived bundles (n=8 bundles for each line). (**d**) Twitch kinetics of 2-week differentiated GM25256 and Fucci derived bundles (n=8 bundles for each line). Data are presented as mean ± SEM.

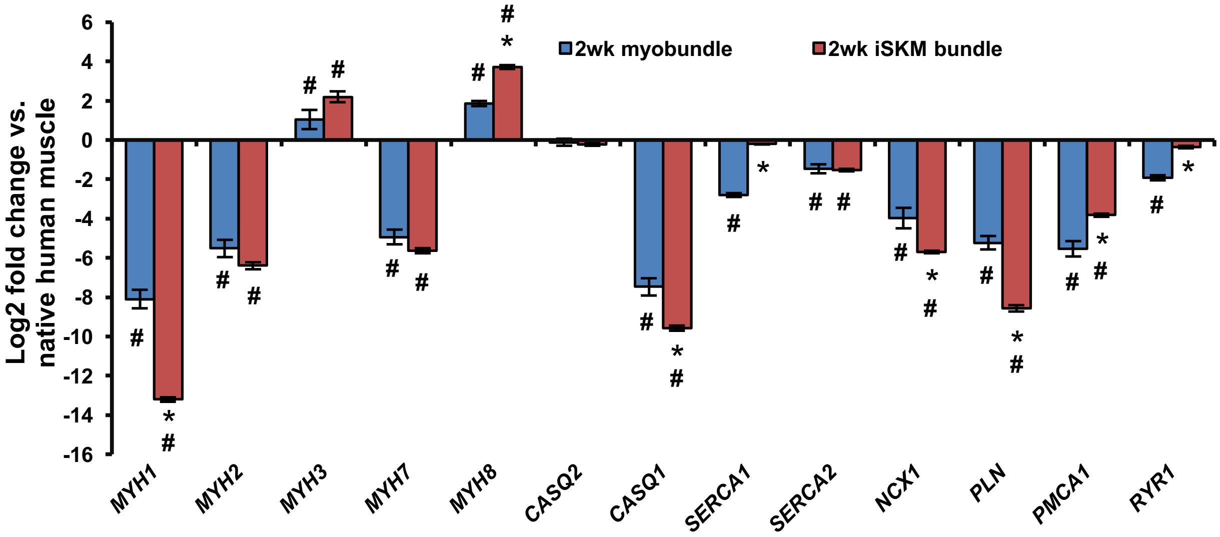

Supplementary Figure 12. Relative mRNA expression levels of different myosin isoforms and calcium handling related genes in 2-week differentiated iSKM bundles, primary human myobundles, and native human muscle. (*P<0.05 vs. 2wk myobundle group, #P<0.05 vs. native human muscle, Tukey-Kramer HSD test; for iSKM bundles, n=8 bundles from 3 differentiations, for primary human myobundles, n=6 bundles from 3 differentiations, for native human muscle, n=3 samples from 3 donors with 4 technical replicates each, data are presented as mean ± SEM).

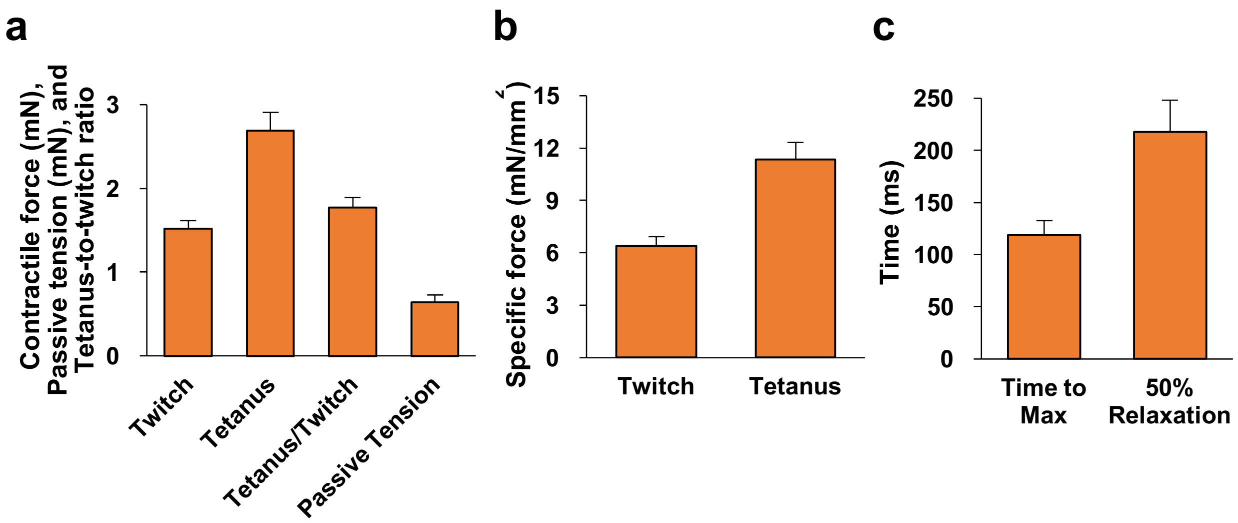

Supplementary Figure 13. Contractile properties of primary human myobundles differentiated for 2 weeks in serum-free iSKM differentiation media. (a) Contractile force, passive tension at 10% elongation and tetanus-to-twitch ratio of 2-week differentiated primary human myobundles. **(b)** Specific force of 2-week primary human myobundles. **(c)** Twitch kinetics of 2-week primary human myobundles (n=6 bundles from 2 differentiations). Data are presented as mean ± SEM.

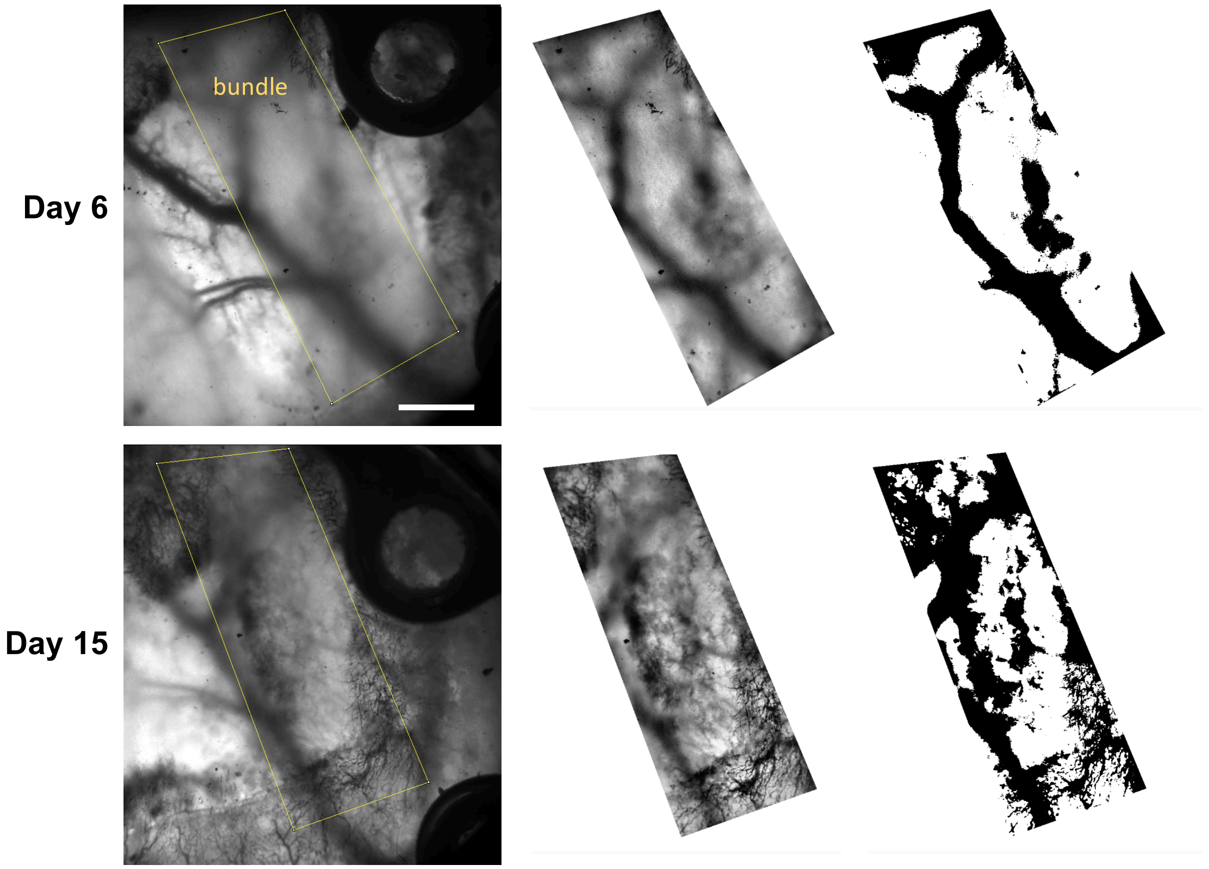

Supplementary Figure 14. Processing and quantification of blood vessel density in iSKM bundles implanted in dorsal skin-fold window chambers in immunocompromised mice. Representative raw (left), cropped/contrast enhanced (middle), and thresholded (right) intravital images used for quantification of blood vessel density in bundle area 6 and 15 days post-implantation. Note increased transparency of the bundle area with time PI. Scale bar, 1mm.

MHCK7-R-GECO

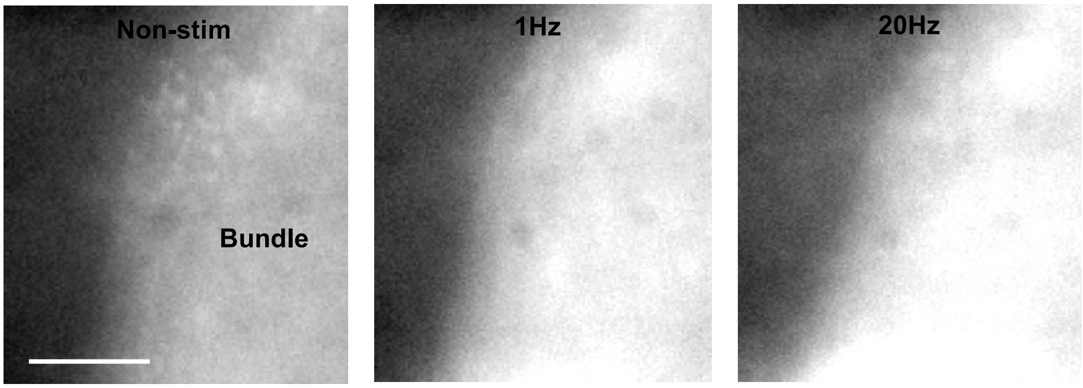

Supplementary Figure 15. Representative images of *ex vivo* recorded R-GECO signals in iSKM bundles implanted for 1 week in mouse TA muscle, shown without stimulation (Non-stim) or during 1Hz and 20Hz stimulation. Scale bar, 50µm.

Supplementary Table 1. Cell culture media and solutions

Media and solutions	Formulation
Neutralizing media	Low glucose DMEM (Thermo) supplemented with DNaseI (20µg/mL, Sigma) and 50% FBS (Hyclone)
Flow buffer	PBS supplemented with 1% FBS (Hyclone) and 0.1% Sodium Azide (Sigma)
Blocking solution	PBS supplemented with 5% chicken serum (Sigma), 0.2%Triton X 100 (Sigma)
Sorting solution	E6 media supplemented with Y27632 (10µM, Tocris), Dox (1µg/mL, Sigma), DNaseI (20µg/mL, Sigma), penicillin G (100 unit/mL, Thermo) and strepomycin (50 µg/mL, Thermo)
Collecting solution	FBS (Hyclone) supplemented with Y27632 (10µM, Tocris), Dox (1µg/mL, Sigma) , penicillin G (100 unit/mL, Thermo) and strepomycin (50 µg/mL, Thermo)
Expansion media (EM)	Low glucose DMEM (Thermo) supplemented with 10% FBS (Hyclone), Fetuin (500x), hEGF (1000x) and Dexamethasone (1000x) from SkGM SingleQuots Kit (Lonza), penicillin G (100 unit/mL, Thermo) and strepomycin (50 µg/mL, Thermo)
Differentiation media (DM)	Low glucose DMEM (Thermo) supplemented with N2 supplement (100x, Thermo), penicillin G (100 unit/mL, Thermo)
Cell/hydrogel mixture	12.5 million cells/mL in expansion media, bovine fibrinogen (4 mg/mL, Sigma), growth factor reduced matrigel (20% v/v, Corning), thrombin (0.2 unit/mg, Sigma)

Supplementary Table 2. Primers used for qRT-PCR.

G3PD F	AGGTCGGAGTCAACGGATTTGG
G3PD R	AGGCTGTTGTCATACTTCTCATGG
T F	CCCGTCTCCTTCAGCAAAGTC
T R	TAAGAGCTGTGATCTCCTCGTTCTG
Tbx6 F	CTCCGTGACAGCCTACCAGA
Tbx6 R	GGTGTGTCTCCGCTCCCATA
Msgn1 F	AGGATGTCTGTCCAGCGGAG
Msgn1 R	AGGAGGTCTGTGAGTTCCCC
Myf5 F	CGCCTGAAGAAGGTCAACCA
Myf5 R	ACATTCGGGCATGCCATCAG
Pax3 F	CCTCAGGTAATGGGACTCCTG
Pax3 R	CCCCCTAAAAAGTCCAAGGCT
Pax7 F	GGAAGCGATTTTTGCCGACT
Pax7 R	TTGTGGCGGATGTGGTTAGG
MyoD F	CGACGGCATGATGGACTACA
MyoD R	TATATCGGGTTGGGGTTCGC
MyoG F	GCCAACCCAGGGGATCAT
MyoG R	CCCGGCTTGGAAGACAATCT
MYH1 F	ATCTAACTGCTGAAAGGTGACC
MYH1 R	TAAGTAAATGGAGTGACAAAG
MYH2 F	GCCGAGTCCCAGGTCAACAAG
MYH2 R	TGAGCAGATCAAGATGTGGCAAAG
MYH3 F	GGAGCAGGACAGAAGATAT
MYH3 R	CCCAGATTGAAACAAAGCA
MYH7 F	CTGTCCAAGTTCCGCAAGGT
MYH7 R	TCATTCAAGCCCTTCGTGCC
MYH8 F	ATTTCCACCAAGAACCCA
MYH8 R	AAAGGATTCTGCCTCTGG
CASQ1 F	ACATTGTGGCCTTCGCAGAG
CASQ1 R	CCATACGCTATCCGCATCAGT
CASQ2 F	TTGCCATCCCCAACAAACCT
CASQ2 R	AGAGTGGGTCTTTGGTGTTCC
RYR1 F	GCAAAGGAGAGACAGGTGCC
RYR1 R	TGGCGATTGATGACAGTGCC
SERCA1 F	GAAGGGAGCACAATGGAGGC
SERCA1 R	CAGGCCAGCACGAAGGAAAT
SERCA2 F	CCAACGTCGGGGAAGTTGTC
SERCA2 R	CGCCGACGTAACAGCCAATA
NCX1 F	AGGCCAACCTGTCTTCAGGA
NCX1 R	AACCACAAGGGCCAGGTTTG
PMCA1 F	AATGGGAACATTACTCTGGGGC
PMCA1 R	AGCATTCACTACATCCATCTGTGT
PLN F	GCTGCCAAGGCTACCTAAAAG
PLN R	GACGTGCTTGTTGAGGCATTT
