# Supplementary material for: Engineering human pluripotent stem cells into a functional skeletal muscle tissue
Source: Nat Commun. 2018 Jan 9;9:126. doi: 10.1038/s41467-017-02636-4 (PMC5760720; doi:10.1038/s41467-017-02636-4)
Supplement: Supplementary file 3 — Description of Additional Supplementary Files [file 41467_2017_2636_MOESM3_ESM.docx]

**Description of Additional Supplementary Files**

File Name: Supplementary Movie 1

Description:

Spontaneous contractions and electrically induced Ca2+ transients in hPSC-derived

myotubes at 2 weeks of monolayer differentiation. Flashes show changes in intracellular calcium levels reported by GCaMP6 fluorescence

File Name: Supplementary Movie 2

Description:

Spontaneous contractions and electrically induced Ca2+ transients in iSKM bundles at 2 weeks of differentiation. Flashes show changes in intracellular calcium levels reported by GCaMP6 fluorescence

File Name: Supplementary Movie 3

Description:

Acetylcholine induced Ca2+ transients in iSKM bundle at 2 weeks of differentiation. Flashes show changes in intracellular calcium levels reported by GCaMP6 fluorescence

File Name: Supplementary Movie 4

Description:

Spontaneous and electrically induced Ca2+ transients in iSKM bundles implanted in window

chambers. Flashes show changes in intracellular calcium levels reported by GCaMP6 fluorescence

File Name: Supplementary Movie 5

Description:

Electrically induced Ca2+ transients in iSKM bundles implanted in TA muscle. Flashes show

changes in intracellular calcium levels reported by R-GECO fluorescence
